# Pan-immune inflammation value: A novel biomarker for cataract

**DOI:** 10.1371/journal.pone.0335713

**Published:** 2025-10-31

**Authors:** Chuan-Xi Wang, Jia-Wei Fan, Jing-Hui Liu, Si-Yu Lin, Jing-Jing Hou, Zheng-Xuan Jiang, Jie Gao

**Affiliations:** 1 Department of Ophthalmology, The Second Affiliated Hospital of Anhui Medical University, Hefei, China; 2 Department of Ophthalmology, Fuyang People’s Hospital, Hangzhou, China; South China University of Technology, CHINA

## Abstract

**Background:**

Inflammation significantly contributes to cataract formation; Pan-immune inflammation value (PIV) is an emerging marker that may reflect systemic inflammation. This study aimed to explore the connection between PIV and cataract formation.

**Methods:**

We analyzed 8,235 participants from the National Health and Nutrition Examination Survey (NHANES) between 2005 and 2008. PIV was log-transformed, and weighted logistic regression, restricted cubic splines (RCS), subgroup, and sensitivity analyses were performed to evaluate associations. The receiver operating characteristic (ROC) curves were used to assess predictive performance.

**Results:**

Cataract prevalence was 6.6%. Higher ln-PIV was positively associated with cataract prevalence [OR=1.35, 95%CI: 1.08–1.69, P = 0.015]. The association was linear, consistent across subgroups, and robust in sensitivity analyses. The ROC analysis indicated that PIV had better predictive ability for cataract than other inflammatory indices.

**Conclusion:**

We found that higher PIV levels were positively associated with cataract prevalence, which highlights the value of inflammatory markers in assessing cataract development and provides new ideas for the clinical detection of cataracts.

## 1. Introduction

The lens is a flat ball with a refractive function that sits between the iris and the vitreous body, and when it becomes cloudy for any reason, it is called a cataract [1]. Studies have shown that age, diabetes and the environment are risk factors for developing cataracts [2–4]. Epidemiological statistics show that cataracts are responsible for 46% of the 180 million people with visual impairment worldwide [5]. Although surgery is an effective treatment for cataracts, the high cost and the shortage of ophthalmologists contribute to cataracts remaining a challenging public health problem [6,7]. Therefore, early diagnosis and intervention of cataracts is of great importance.

The inflammatory response plays a key role in cataract development [8]. Recently, many inflammatory markers have been developed using complete blood counts that are valuable in predicting disease onset and clinical outcome [9], and pan-immune inflammation value (PIV) is one of them. Compared with systemic immune-inflammation index (SII: platelet count × neutrophil/lymphocyte) and PLR (platelet/ lymphocyte), PIV may be biologically superior because it additionally incorporates monocytes, which helps to better reflect immune dysregulation and inflammatory cascade reactions [10,11]. Previous studies have shown an association with the development of stroke, abdominal aortic calcification, COPD and cancer [12–16]. However, no studies have investigated the relationship between PIV and cataracts.

To address this gap, we utilized the National Health and Nutrition Examination Survey (NHANES) database, which includes samples from individuals aged 20 and older, to examine the relationship between PIV and cataracts.

## 2. Method

### 2.1. Study population

NHANES is a biennial national survey conducted by the Centers for Disease Control and Prevention (CDC) in the United States to evaluate the health and nutritional status of American citizens. Participation requires individuals to provide informed consent. Human studies in NHANES were approved by the Ethical Review Board of the National Centre for Health Statistics. Informed consent was obtained from all participants.

**Fig 1:**
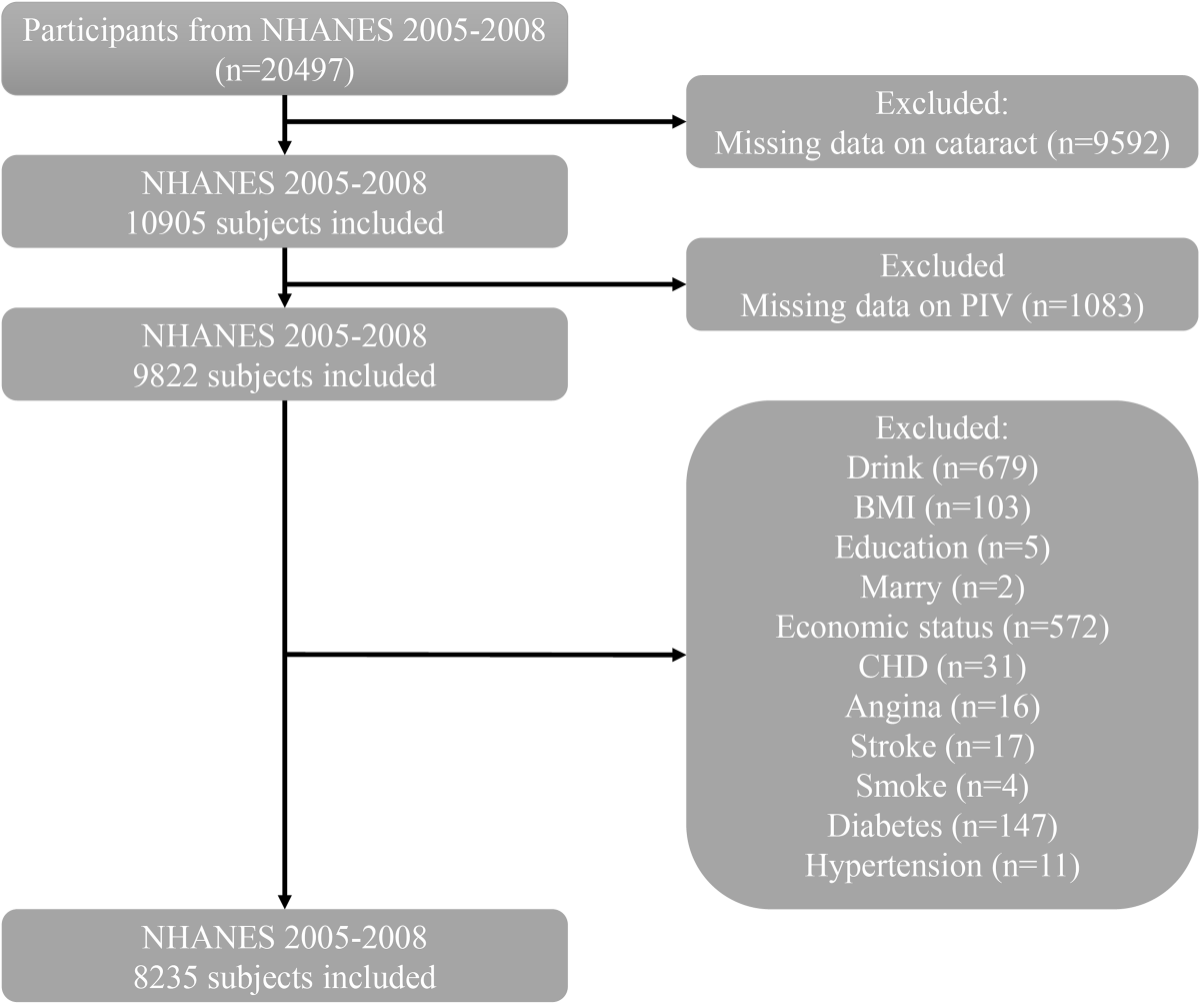
Flowchart of participant selection from NHANES 2005-2008. Abbreviations: NHANES: National Health and Nutrition Examination Survey; PIV: pan-immune inflammation value; BMI: body mass index; CHD: coronary heart disease.

We initially obtained 20,497 subjects for the two cycles 2005–2008, excluding participants: 1) did not have complete cataract information; 2) did not have complete PIV data. Classification of variables such as education level, marital status, smoking, drinking, hypertension, diabetes, and cardiovascular disease was primarily based on standardized NHANES questionnaires. Participants with clear responses were included, while those who responded “refused”, don’t know or “missing” were excluded. For BMI and economic status, individuals with missing values were excluded from the analysis. More detailed information can be found in S1 and S5 Tables. The specific flowchart is shown in Fig 1. Ultimately, we included 8,235 participants.

### 2.2. Calculation of PIV

**Fig 2:**
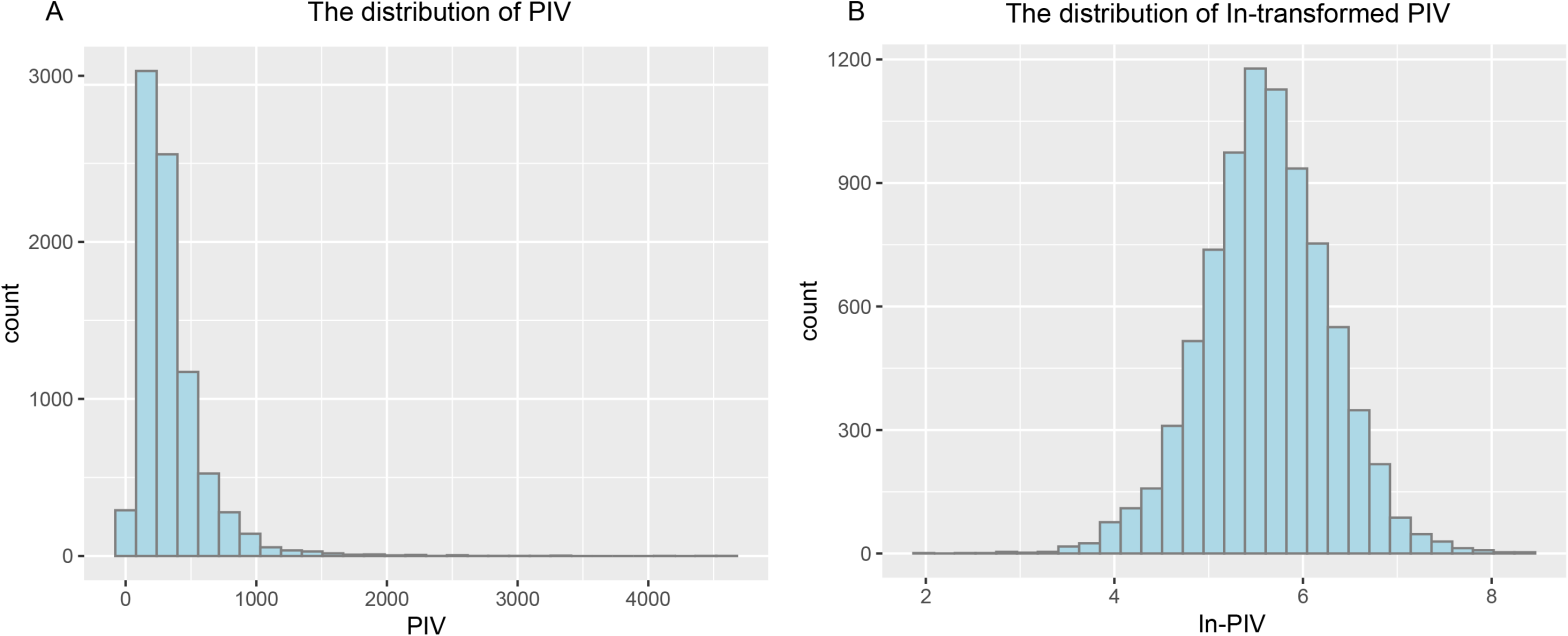
The map of PIV distribution (A), and the distribution of PIV after ln-transformation (B). Abbreviation: PIV: pan-immune inflammation value.

PIV combines neutrophils (Neu), lymphocytes (Lym), monocytes (Mono) and platelets (PLT) in a complete blood count and is calculated as: Neu (10^9^/L) *PLT (10^9^/L) *Mono (10^9^/L)/Lym (10^9^/L) [17,18]. It has been converted to ln (PIV) to fit a normal distribution (Fig 2).

### 2.3. Definition of cataract

Consistent with other studies, we used cataract surgery instead of diagnosis [19,20]. The researcher asked participants: “Have you had a cataract surgery?” Those who answered ‘Yes’ were diagnosed with cataracts. Otherwise, they did not have cataracts.

### 2.4. Covariates

In our synthesis of earlier research, we incorporated a range of demographic factors—including age, gender, ethnicity, educational background, marital status, and economic status—alongside body mass index (BMI), lifestyle choices such as smoking and alcohol use, and coexisting health conditions like hypertension, diabetes, coronary artery disease, angina, and stroke as covariates. Demographic information was obtained from the questionnaire, and BMI was further categorized as (<18.5, 18.5–25, > 25 kg/m^2^). Smoking, alcohol consumption, coronary heart disease (CHD), angina and stroke were self-reported. Participants were classified as having hypertension if they had received a diagnosis from a physician, were currently on antihypertensive medication, or exhibited a mean systolic blood pressure of 140 mmHg or higher or a mean diastolic blood pressure of 90 mmHg or higher. Diabetes mellitus diagnostic criteria included: 1) receiving a diabetes diagnosis from a physician, 2) taking insulin and glucose-lowering medication, and 3) having glycated hemoglobin ≥6.5%.

### 2.5. Data analysis

Physical examination and laboratory data were collected at the Mobile Examination Center (MEC). NHANES provides MEC examination weights (WTMEC2YR) to account for the complex survey design and ensure nationally representative estimates. As for the four years 2005–2008 were the two-year WTMEC2YR/2. We divided participants into cataract and non-cataract groups, with categorical and continuous variables expressed as percentages and means (standard deviations). We used chi-squared and T-tests to compare baseline characteristics between the two groups. The association between ln-PIV and risk of cataract development was investigated using weighted logistic regression: model 1: original model; model 2: age, sex, race; model 3; further adjusted for education, economic level, marital status, BMI, smoking, alcohol consumption, hypertension, diabetes, CHD, angina and stroke. Smoothed curve fitting and RCS analyses assessed the dose-response relationship between ln-PIV and cataract prevalence. Subgroup and sensitivity analyses were conducted to evaluate the robustness of the findings. Finally, we plotted ROC curves and calculated the area under the curve to assess and compare the predictive value of PIV for cataract occurrence. Data were analyzed using R Studio (version 4.3.1) and Empower Stats (version 2.0). p < 0.05 was defined as significant.

## 3. Results

### 3.1. Basic traits of the participants

The research involved 8235 individuals, with a cataract incidence rate of 6.6%. Table 1 shows the population characteristics of the cataract and non-cataract groups. Older age, female sex, lower education and economic level, as well as comorbidities including diabetes, hypertension, stroke, angina, and coronary heart disease, showed significant associations with cataract prevalence.

**Table 1:** Characteristics of participants stratified by cataract from NHANES 2005–2008.

	All	Non-cataract	Cataract	*p*-value
Number	8235	7502 (93.4)	733 (6.6)	
Gender (N, %)				<0.001
Male	4007 (48.2)	3669 (48.9)	338 (38.3)	
Female	4228 (51.8)	3833 (51.1)	395 (61.7)	
Age [years, mean (SD)]	46.40 (16.49)	44.59 (15.23)	72.12 (11.59)	<0.001
Ethnicity (N, %)				<0.001
Mexican American	1513 (7.9)	1458 (8.3)	55 (2.5)	
Other Hispanic	577 (3.9)	541 (4.1)	36 (2.2)	
Non-Hispanic White	4158 (72.7)	3629 (71.7)	529 (85.9)	
Non-Hispanic Black	1683 (10.3)	1587 (10.7)	96 (5.7)	
Other	304 (5.1)	287 (5.2)	17 (3.6)	
Education (N, %)				<0.001
Less Than 9th Grade	948 (5.8)	798 (5.3)	150 (13.2)	
9–11th Grade	1349 (12.1)	1231 (11.9)	118 (14.4)	
Highschool graduate or equivalent	1980 (24.6)	1789 (24.3)	191 (29.4)	
Some College or AA degree	2268 (30.6)	2108 (31.0)	160 (25.0)	
College graduate or above	1690 (26.8)	1576 (27.4)	114 (18.0)	
BMI (N, %)				0.127
< 25 kg/m ^2^	2443 (32.4)	2216 (32.4)	227 (33.2)	
25–30 kg/m ^2^	2852 (33.8)	2581 (33.6)	271 (36.9)	
> 30 kg/m ^2^	2940 (33.7)	2705 (34.0)	235 (29.8)	
Economic level (N, %)				<0.001
< 1	1524 (12.0)	1422 (12.2)	102 (8.5)	
1-3	3467 (35.4)	3035 (33.8)	432 (58.1)	
> 3	3244 (52.7)	3045 (54.0)	199 (33.3)	
Marital status (N, %)				<0.001
Married or living with a partner	5149 (65.9)	4759 (66.7)	390 (54.7)	
Unmarried or other	3086 (34.1)	2743 (33.3)	343 (45.3)	
Alcohol consumption (N, %)				<0.001
Yes	5777 (74.9)	5340 (75.9)	437 (61.0)	
No	2458 (25.1)	2162 (24.1)	296 (39.0)	
Smoking status (N, %)				<0.001
Never	4292 (51.8)	3947 (52.1)	345 (47.3)	
Now	1837 (23.4)	1767 (24.4)	70 (9.5)	
Former	2106 (24.8)	1788 (23.5)	318 (43.2)	
Hypertension (N, %)				<0.001
Yes	2742 (29.7)	2303 (27.7)	439 (58.8)	
No	5493 (70.3)	5199 (72.3)	294 (41.2)	
Diabetes (N, %)				<0.001
Yes	1136 (9.9)	927 (8.8)	209 (25.3)	
No	7099 (90.1)	6575 (91.2)	524 (74.7)	
CHD (N, %)				<0.001
Yes	323 (3.1)	237 (2.6)	86 (11.6)	
No	7912 (96.9)	7265 (97.4)	647 (88.4)	
Angina (N, %)				<0.001
Yes	224 (2.2)	165 (1.8)	59 (7.4)	
No	8011 (97.8)	7337 (98.2)	674 (92.6)	
Stroke (N, %)				<0.001
Yes	299 (2.7)	208 (2.0)	91 (12.7)	
No	7936 (97.3)	7294 (98.0)	642 (87.3)	
PIV	344.65 (256.11)	339.38 (247.50)	419.65 (348.42)	<0.001

Abbreviations: SD: Standard Deviation; BMI: body mass index; CHD: coronary heart disease.

### 3.2. Association between PIV and incident cataract

**Fig 3:**
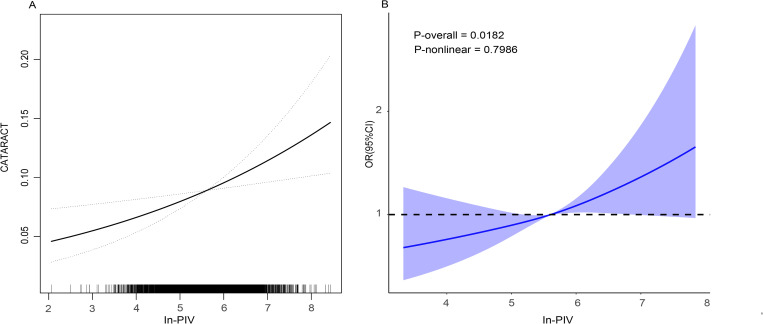
The linear relationship between ln-PIV and risk of cataract. Smoothed curve fitting (A). Restricted cubic splines (B). Analyses were adjusted for age, gender, ethnicity, educational background, marital status, economic status, BMI, smoking and alcohol use, hypertension, diabetes, coronary heart disease, angina, and stroke. Abbreviations: PIV: pan-immune inflammation value; BMI: body mass index; OR: odd rations; CI: confidence interval.

Table 2 presents findings from the weighted logistic regression, indicating a positive correlation between ln-PIV and cataract prevalence in models 1, 2, and 3. When ln-PIV was categorized by quartiles, a higher ln-PIV was associated with a greater prevalence of cataracts. Smoothed curve fitting and RCS analysis showed a linear association between ln-PIV and cataracts (Fig 3).

**Table 2:** Weighted multivariate logistic regression analysis of ln-PIV and cataract.

Variables	Model 1	Model 2	Model 3
	OR (95% CI) *P*-value	OR (95% CI) P-value	OR (95% CI) P-value
ln-PIV	1.57 (1.33, 1.85), < 0.001	1.35 (1.09,1.67), 0.008	1.35 (1.08,1.69),**0.015**
Ln-PIV category analysis			
Q1	ref	ref	ref
Q2	1.15 (0.83,1.61), 0.383	1.21 (0.83,1.76), 0.299	1.25 (0.79,1.97), 0.294
Q3	1.36 (0.99,1.89), 0.060	1.30 (0.90,1.88), 0.157	1.31 (0.86,1.99), 0.168
Q4	1.97 (1.46,2.67), < 0.001	1.57 (1.08,2.29), **0.021**	1.58 (1.04,2.42), **0.037**

Model 1: unadjusted.

Model 2: Model 1 + age, gender and ethnicity.

Model 3: Model2 + educational level, marital status, BMI, economic level, smoking status, alcohol consumption, hypertension, CHD, diabetic, angina and stroke.

Abbreviations: BMI: body mass index; OR: odds ratio; CI: confidence interval. CHD: Coronary heart disease.

### 3.3. Subgroup analysis

**Fig 4:**
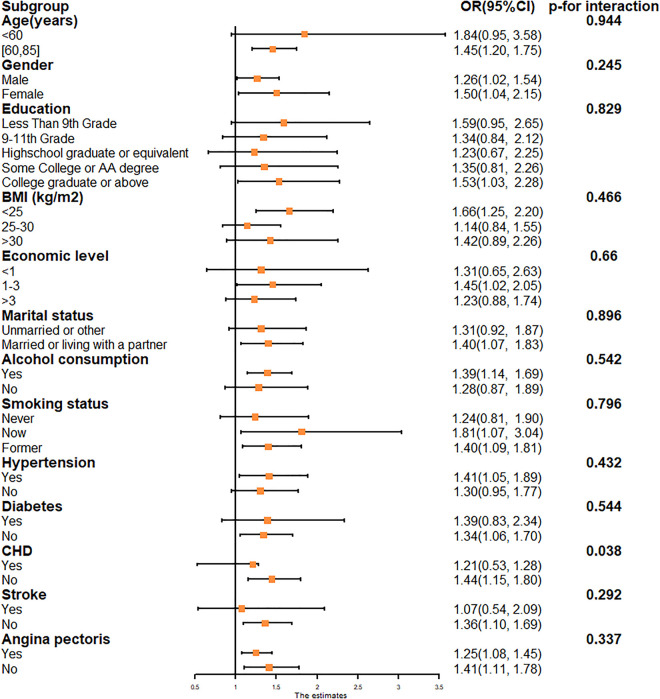
Forest maps for subgroup analyses. Age, gender, ethnicity, educational background, marital status, economic status, BMI, smoking and alcohol use, hypertension, diabetes, CHD, angina, and stroke were adjusted in the subgroup analyses. Abbreviations: PIV: pan-immune inflammation value; BMI: body mass index; OR: odds ratio; CI: confidence interval. CHD: coronary heart disease.

**Fig 5:**
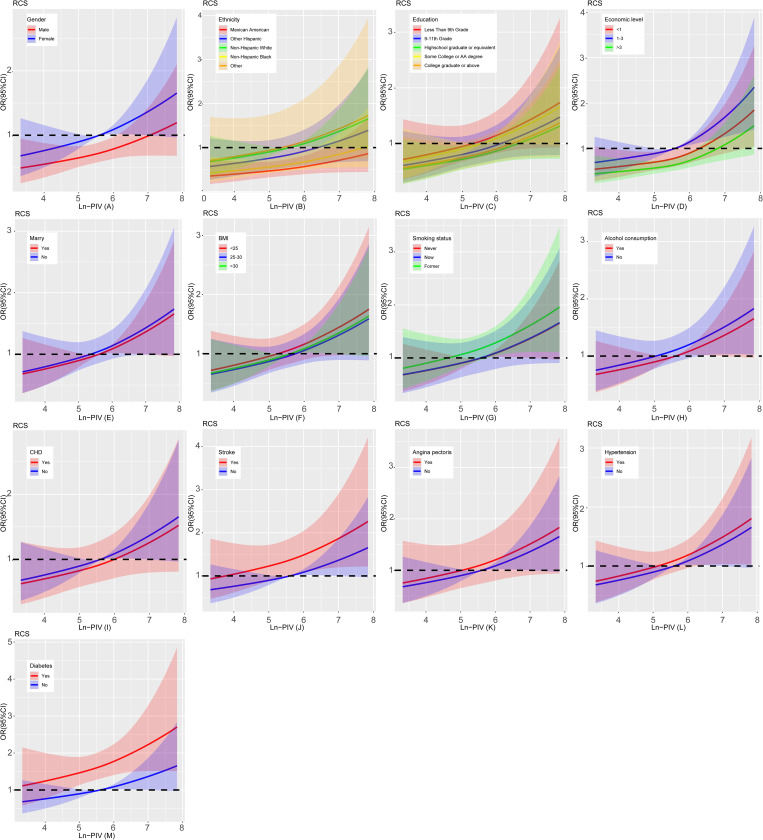
Stratified RCS analysis. Adjusted for age, gender, ethnicity, educational level, marital status, BMI, economic level, smoking status, alcohol consumption, hypertension, CHD, diabetic, angina and stroke.

Stratified analyses by key covariates demonstrated that the positive association between ln-PIV and cataract persisted across all subgroups, with a potential modifying effect of coronary heart disease (Fig 4). Stratified RCS results show linear relationship between Ln-PIV and cataract prevalence is stable across subgroups (Fig 5).

### 3.4. Sensitivity analyses

First, multivariate logistic analyses were performed using unweighted data (The original NHANES sample data before applying survey weights). Secondly, we deleted the top and bottom 1% of PIV values; the outcomes of logistic regression analyses and RCS indicated that the association stayed linearly positive (S2, S3 Tables and S1 Fig).

### 3.5. ROC curve

**Fig 6:**
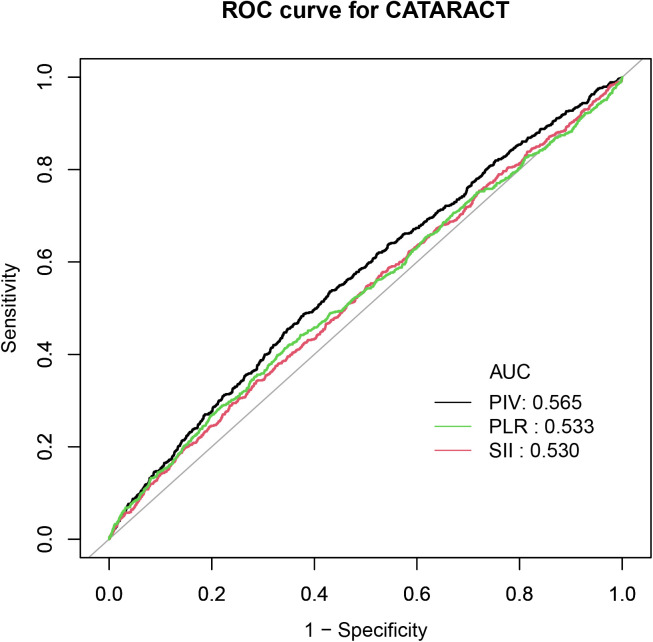
Receiver operating characteristic curve (ROC) of PIV, SII, and PLR. Abbreviations: AUC: area under the curve; PIV: pan-immune inflammation value; SII: systemic immune-inflammation index; PLR: platelet to lymphocyte ratio.

The ROC analysis indicated that PIV had a stronger predictive ability than SII and PLR (Fig 6). Furthermore, DeLong’s test confirmed the superiority of PIV over SII and PLR in predicting cataracts (p < 0.01), with detailed results provided in Supplementary S4 Table.

## 4. Discussion

The study preliminarily analyzed the relationship between PIV and cataract prevalence utilizing the NHANES. The findings indicated a positive correlation between ln-PIV and cataract prevalence, which remained stable after removing extreme values and analyzing with unweighted data. Subgroup analyses showed a significant interaction between the two associations only in the coronary heart disease strata.

Inflammatory markers derived from whole blood cells are a hot topic of research. Huang and Li et al. discovered a positive association between SII and cataract risk [21,22]. Hou et al. also found that elevated NLR was associated with a higher prevalence of cataract [23]. And our preliminary study further showed the association between PIV and cataract. The ROC curve outcomes indicated that PIV showed better discriminatory ability for cataract prevalence compared to SII and PLR.

Neutrophils, lymphocytes, monocytes and platelets are closely related to the level of systemic inflammation. When an inflammatory response occurs, the number of neutrophils increases to clear inflammatory pathogens [24]. Neutrophils and monocytes are stimulated to release inflammatory factors such as Interleukin-1, IL-8 and Tumor Necrosis Factor necrosis factor-a (TNF-a), leading to high levels of inflammation in the eye [25,26]. Lymphocytes have the opposite role to neutrophils in that they regulate the inflammatory response and act as an indicator of physiological stress [27,28]. Platelets play an essential role in thrombosis. Recent studies have shown that they can mediate inflammation, for example, when megakaryocytes are stimulated by platelets and release p-selectin to mediate leukocyte rolling [29,30]. And activated platelets can form particles in the circulation that promote monocyte aggregation towards the inflamed endothelium [31,32].

Persistent inflammation is closely related to the progression of cataracts. This preliminary research enhances the correlation between inflammatory indicators and cataract susceptibility, as elevated PIV indicates increased levels of systemic inflammation [33]. In the inflammatory state, inflammatory cells secrete large amounts of inflammatory factors, leading to increased levels of intraocular inflammation, and lens proteins are denatured and aggregated under prolonged stimulation by inflammatory factors [34,35]. In addition, inflammation is often accompanied by oxidative stress, and free radicals released by inflammatory cells may also alter lens proteins, potentially influencing cataract formation [36].

In our preliminary study, PIV showed a slightly higher predictive value for cataract development (AUC = 0.565) than PLR (AUC = 0.533) and SII (AUC = 0.530). Unlike PLR and SII, PIV incorporates monocytes in addition to neutrophils, platelets, and lymphocytes, providing a more comprehensive reflection of systemic inflammation and the oxidative stress environment relevant to cataract. Monocytes play a key role in the microinflammatory processes of cataract by secreting pro-inflammatory factors and promoting lens protein denaturation. By integrating multiple immune cell types, PIV also reduces the random variability inherent to single-cell indices, improving stability. Nevertheless, the AUC is barely above 0.5, and the confidence intervals in the study are very wide. This shows a possible overall trend of association between systemic inflammation and cataract development. The causal relationship and potential clinical utility of PIV require validation in future large-scale longitudinal studies.

These results indicate that PIV may have potential clinical utility due to its low cost and accessibility. Compared with NLR and SII, PIV could better capture systemic inflammatory status. However, its complexity and lack of standardized cut-offs currently limit clinical application. As this is an observational study, the findings reflect association rather than causation. Future studies should focus on standardizing PIV, validating it longitudinally, and evaluating its potential role in cataract prevention to determine its added clinical value.

The inclusion of a representative sample and using two sensitivity analyses further increase the credibility and universality of the findings. However, the preliminary research possesses several constraints.

A key limitation of this preliminary study is the reliance on self-reported cataract surgery history as a proxy for cataract diagnosis. This approach likely underestimates the true prevalence, as individuals with untreated cataracts are misclassified as “non-cataract.” Prior research has shown that self-reported data on eye diseases and surgical history can substantially underestimate disease burden, particularly in diverse populations. This limitation may also introduce potential selection bias by disproportionately excluding individuals with operable but untreated cataracts, leading to an underestimation of cataract-related associations [37]. Due to the NHANES database lacks standardized clinical ophthalmic examinations, precise quantification of this bias is challenging. However, population-based studies provide some reference: the proportion of operated cataracts among operable cases varies widely, with a median of approximately 60.5% in high-income countries compared to only 14.8% in low-income countries. This suggests that in high-income settings, self-reported surgery may capture only about 60% of operable cases, whereas in low-income settings the underestimation could exceed 85% [38,39]. Given this constraint, we emphasize that future work should incorporate objective ophthalmic examinations to capture both treated and untreated cases, and we caution readers to interpret our findings carefully when generalizing to broader populations.

Furthermore, the NHANES dataset does not provide information on the timing of cataract surgery relative to PIV measurement, preventing assessment of temporal or causal relationships. As PIV reflects inflammatory status at a single time point, it may be influenced by transient factors, such as acute infections, and therefore may not accurately represent chronic inflammation. Consequently, our findings should be interpreted as a cross-sectional association between PIV and history of cataract surgery, rather than an indicator of long-term inflammatory status. Future longitudinal studies with repeated measurements of inflammatory markers are warranted to better elucidate the relationship between PIV and cataract development.

## 5. Conclusion

This preliminary research indicated a positive association between PIV and cataract prevalence, supporting the notion that systemic inflammation may be related to cataract. Further prospective and mechanistic studies are needed to validate these findings and explore the potential pathways underlying the observed association.

## Supporting information

S1 Table Inclusion and exclusion criteria for study variables. (DOCX)

S2 Table Unweighted multivariate logistic regression analysis of PIV and cataract. (DOCX)

S3 Table Weighted multivariate logistic regression analysis of PIV and cataract after deleting extreme values. (DOCX)

S4 Table Comparison of AUCs Between inflammation markers using DeLong’s Test. (DOCX)

S5 Table Differences in baseline characteristics between included and excluded participants. (DOCX)

S1 Fig Linear relationship between ln-PIV and risk of cataract after excluding extreme values. (DOCX)
