# Supplementary material for: Pan-immune inflammation value: A novel biomarker for cataract
Source: PLoS One. 2025 Oct 31;20(10):e0335713. doi: 10.1371/journal.pone.0335713 (PMC12578218; doi:10.1371/journal.pone.0335713)
Supplement: S1 Table — (DOCX) [file pone.0335713.s001.docx]

**Table S1.** Inclusion and Exclusion Criteria for Study Variables**.**

| **Variable** | **Inclusion Criteria** | **Exclusion Criteria** | **Question** |
| --- | --- | --- | --- |
| Drink | Yes / No | Refused / Don’t know / Missing | Had at least 12 alcohol drinks in 1 year? |
| BMI (kg/m²) | 12.5 – 73.4 | Missing | Body Mass Index |
| Education Level | • Less than 9th grade • 9–11th grade • High school / GED • Some college / AA degree • College graduate or above | Refused / Don’t know / Missing | Education Level |
| Marital Status | • Married • Widowed • Divorced • Separated • Never married • Living with partner | Refused / Don’t know / Missing | Marital Status |
| Economic Status | 0–5 | Missing | Ratio of family income to poverty |
| CHD | Yes / No | Refused / Don’t know / Missing | Ever told you had coronary heart disease? |
| Angina | Yes / No | Refused / Don’t know / Missing | Ever told you had angina/angina pectoris? |
| Stroke | Yes / No | Refused / Don’t know / Missing | Ever told you had a stroke? |
| Smoke | Yes / No | Refused / Don’t know / Missing | 1. Smoked at least 100 cigarettes in life? 2. Do you now smoke cigarettes? |
| Diabetes | Meet one of the conditions or complete information: | Refused / Don’t know / Missing | • Doctor told you have diabetes  • Taking insulin now • Take diabetic pills to lower blood sugar • Hemoglobin ≥ 6.5% |
| Hypertension | Meet one of the conditions or complete information: | Refused / Don’t know / Missing | • Ever told you had high blood pressure  • Now taking prescribed medicine for HBP  • Mean systolic BP ≥ 140 mmHg  • Mean diastolic BP ≥ 90 mmHg |
