# Supplementary material for: Pan-immune inflammation value: A novel biomarker for cataract
Source: PLoS One. 2025 Oct 31;20(10):e0335713. doi: 10.1371/journal.pone.0335713 (PMC12578218; doi:10.1371/journal.pone.0335713)
Supplement: S2 Table — (DOCX) [file pone.0335713.s002.docx]

**Table S2.** Unweighted multivariate logistic regression analysis of PIV and cataract.

| **Variables** | **Model 1** | **Model 2** | **Model 3** |
| --- | --- | --- | --- |
|  | OR (95% CI) *P*-value | OR (95% CI) P-value | OR (95% CI) P-value |
| ln-PIV | 1.42(1.27,1.60), <0.001 | 1.23(1.07,1.41), 0.003 | 1.22(1.06,1.40), **0.005** |
| ln-PIV category analysis | | | |
| Q1 | ref | ref | ref |
| Q2 | 1.19(0.94,1.50), 0.154 | 1.16(0.88,1.53), 0.282 | 1.14(0.87,1.51), 0.341 |
| Q3 | 1.39(1.10,1.74), 0.005 | 1.25(0.95,1.64), 0.111 | 1.21(0.92,1.59), 0.175 |
| Q4 | 1.81(1.45,2.25), <0.001 | 1.42(1.09,1.85), 0.010 | 1.39(1.06,1.82), **0.018** |

Model 1: unadjusted

Model 2: Model 1+age, sex and ethnicity

Model 3: Model2 + educational level, marital status, BMI, economic level, smoking status, alcohol consumption, hypertension, CHD, diabetic, angina and stroke.

Abbreviations: BMI: body mass index; OR: odds ratio; CI: confidence interval. CHD: Coronary heart disease
