# Supplementary material for: Pan-immune inflammation value: A novel biomarker for cataract
Source: PLoS One. 2025 Oct 31;20(10):e0335713. doi: 10.1371/journal.pone.0335713 (PMC12578218; doi:10.1371/journal.pone.0335713)
Supplement: S3 Table — (DOCX) [file pone.0335713.s003.docx]

**Table S3.** Weighted multivariate logistic regression analysis of PIV and cataract after deleting extreme values.

| **Variables** | **Model 1** | **Model 2** | **Model 3** |
| --- | --- | --- | --- |
|  | OR (95% CI) *P*-value | OR (95% CI) P-value | OR (95% CI) P-value |
| ln PIV | 1.51(1.28,1.79), <0.001 | 1.30(1.03,1.63),0.026 | 1.31(1.02,1.68),**0.038** |
| ln PIV category analysis | | | |
| Q1 | ref | ref | ref |
| Q2 | 1.15(0.83,1.60),0.399 | 1.18(0.80,1.73),0.385 | 1.21(0.76, 1.94),0.364 |
| Q3 | 1.36(0.98,1.87),0.064 | 1.26(0.86,1.85),0.215 | 1.28(0.83, 1.97),0.218 |
| Q4 | 1.87(1.40,2.51), <0.001 | 1.48(1.02,2.16),0.040 | 1.50(1.02,2.31),**0.042** |

Model 1: unadjusted

Model 2: Model 1+age, sex and ethnicity

Model 3: Model2 + educational level, marital status, BMI, economic level, smoking status, alcohol consumption, hypertension, CHD, diabetic, angina and stroke.

Abbreviations: BMI: body mass index; OR: odds ratio; CI: confidence interval. CHD: Coronary heart disease
