# Supplementary material for: Pan-immune inflammation value: A novel biomarker for cataract
Source: PLoS One. 2025 Oct 31;20(10):e0335713. doi: 10.1371/journal.pone.0335713 (PMC12578218; doi:10.1371/journal.pone.0335713)
Supplement: S4 Table — (DOCX) [file pone.0335713.s004.docx]

**Table S4.** Comparison of AUCs Between Inflammation Markers Using DeLong's Test

| Disease | Comparison | AUC of Marker 1 | AUC of Marker 2 | Z Statistic (DeLong Test) | *p* |
| --- | --- | --- | --- | --- | --- |
| Cataract | PIV vs. SII | 0.565 | 0.530 | 5.929 | <0.001 |
|  | PIV vs. PLR | 0.565 | 0.533 | 2.638 | 0.008 |
|  |  |  |  |  |  |

Note: This table presents the results of DeLong’s test for comparing AUCs between PIV and inflammation markers (SII and PLR) for identifying cataract. Statistically significant differences (*p* < 0.05) suggest that the composite ratio provides superior diagnostic performance.
