# Supplementary material for: Pan-immune inflammation value: A novel biomarker for cataract
Source: PLoS One. 2025 Oct 31;20(10):e0335713. doi: 10.1371/journal.pone.0335713 (PMC12578218; doi:10.1371/journal.pone.0335713)
Supplement: S5 Table — (DOCX) [file pone.0335713.s005.docx]

**Table S5.** Differences in Baseline Characteristics Between Included and Excluded Participants.

|  | All | Exclude | Include | *p-value* |
| --- | --- | --- | --- | --- |
| Number | 10905 | 2670 | 8235 |  |
| **Age [years, mean (SD)]** | 46.68 (16.87) | 47.51 (18.50) | 46.48 (16.47) | 0.095 |
| **Gender (N, %)** |  |  |  | 0.619 |
| Male | 5289 (48.1) | 1275 (47.5) | 4014 (48.3) |  |
| Female | 5616 (51.9) | 1395 (52.5) | 4221 (51.7) |  |
| **Education (N, %)** |  |  |  | <0.001 |
| Less Than 9th Grade | 1439 (6.7) | 492 (10.5) | 947 (5.8) |  |
| 9-11th Grade | 1815 (12.5) | 466 (14.1) | 1349 (12.1) |  |
| Highschool graduate or equivalent | 2645 (25.3) | 657 (27.7) | 1988 (24.7) |  |
| Some College or AA degree | 2923 (30.0) | 661 (27.6) | 2262 (30.6) |  |
| College graduate or above | 2083 (25.6) | 394 (20.2) | 1689 (26.8) |  |
| **Marital status (N, %)** |  |  |  | 0.001 |
| Unmarried or other | 4296 (35.2) | 1206 (40.5) | 3090 (34.0) |  |
| Married or living with a partner | 6609 (64.8) | 1464 (59.5) | 5145 (66.0) |  |
| **Economic level (N, %)** |  |  |  | <0.001 |
| <1 | 2137 (12.9) | 614 (17.2) | 1523 (11.9) |  |
| 1-3 | 4705 (36.7) | 1235 (42.6) | 3470 (35.4) |  |
| >3 | 4063 (50.3) | 821 (40.2) | 3242 (52.7) |  |
| **Ethnicity (N, %)** |  |  |  | <0.001 |
| Mexican American | 2034 (8.2) | 531 (9.4) | 1503 (7.9) |  |
| Other Hispanic | 817 (4.1) | 236 (4.9) | 581 (4.0) |  |
| Non-Hispanic White | 5254 (70.6) | 1098 (61.8) | 4156 (72.7) |  |
| Non-Hispanic Black | 2348 (11.4) | 656 (15.7) | 1692 (10.4) |  |
| Other | 452 (5.7) | 149 (8.3) | 303 (5.1) |  |
| **Alcohol consumption (N, %)** |  |  |  | 0.003 |
| Yes | 7606 (74.0) | 1827 (70.5) | 5779 (74.9) |  |
| No | 3299 (26.0) | 843 (29.5) | 2456 (25.1) |  |
| **CHD (N, %)** |  |  |  | 0.023 |
| Yes | 467 (3.4) | 148 (4.4) | 319 (3.1) |  |
| No | 10438 (96.6) | 2522 (95.6) | 7916 (96.9) |  |
| **Angina (N, %)** |  |  |  | 0.041 |
| Yes | 326 (2.3) | 104 (3.1) | 222 (2.1) |  |
| No | 10579 (97.7) | 2566 (96.9) | 8013 (97.9) |  |
| **Stroke (N, %)** |  |  |  | <0.001 |
| Yes | 455 (3.1) | 155 (4.5) | 300 (2.8) |  |
| No | 10450 (96.9) | 2515 (95.5) | 7935 (97.2) |  |
| **BMI (kg/m^2^)** |  |  |  | 0.407 |
| <25 | 3202 (32.8) | 765 (34.6) | 2437 (32.4) |  |
| [25,30] | 3546 (33.5) | 696 (32.3) | 2850 (33.8) |  |
| >30 | 3680 (33.7) | 732 (33.0) | 2948 (33.9) |  |
| **Smoking status (N, %)** |  |  |  | 0.113 |
| Never | 5754 (52.2) | 1462 (53.7) | 4292 (51.8) |  |
| Now | 2411 (23.4) | 577 (23.7) | 1834 (23.3) |  |
| Former | 2740 (24.5) | 631 (22.6) | 2109 (24.9) |  |
| **CHF (N, %)** |  |  |  | <0.001 |
| Yes | 406 (2.5) | 155 (4.3) | 251 (2.1) |  |
| No | 10499 (97.5) | 2515 (95.7) | 7984 (97.9) |  |
| **Hypertension (N, %)** |  |  |  | 0.560 |
| Yes | 4307 (35.0) | 1060 (35.7) | 3247 (34.8) |  |
| No | 6598 (65.0) | 1610 (64.3) | 4988 (65.2) |  |
| **Cataract (N, %)** |  |  |  | 0.002 |
| Yes | 1071 (7.0) | 337 (9.0) | 734 (6.6) |  |
| No | 9834 (93.0) | 2333 (91.0) | 7501 (93.4) |  |

**Abbreviations:** SD: Standard Deviation; CHD: Coronary heart disease; CHF: congestive heart failure; BMI: body mass index;
