# Supplementary material for: Pan-immune inflammation value: A novel biomarker for cataract
Source: PLoS One. 2025 Oct 31;20(10):e0335713. doi: 10.1371/journal.pone.0335713 (PMC12578218; doi:10.1371/journal.pone.0335713)
Supplement: S1 Fig — (DOCX) [file pone.0335713.s006.docx]

Figure S1. Linear relationship between ln-PIV and risk of cataract after excluding extreme values.
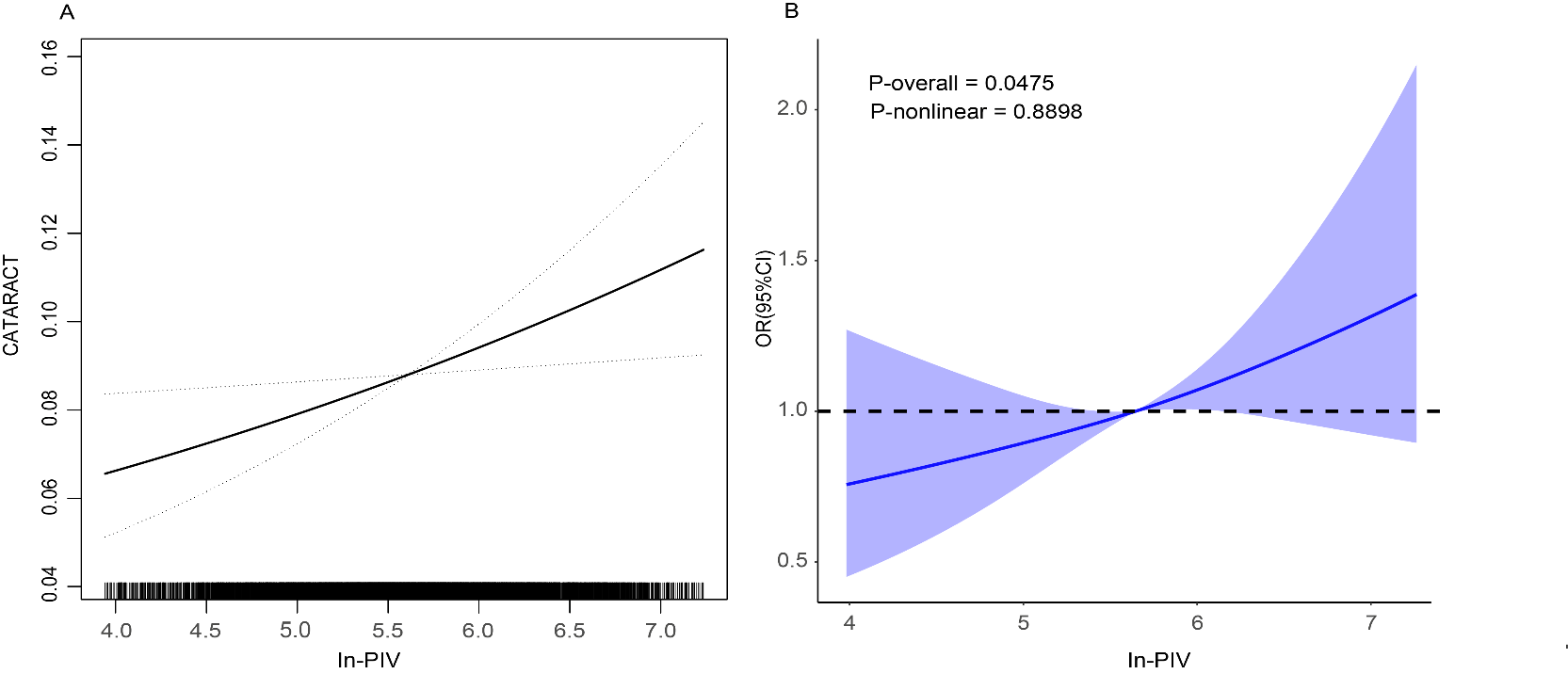


Smoothed curve fitting (A). Restricted cubic splines (B). Analyses were adjusted for age, gender, ethnicity, educational background, marital status, economic status, BMI, smoking and alcohol use, hypertension, diabetes, coronary heart disease, angina, and stroke.

Abbreviations: PIV: pan-immune inflammation value; BMI: body mass index; OR: odd rations; CI: confidence interval.
